# Multi-Temperature Crystallography of Polyamine Biosynthesis Enzymes Reveals Differing Active Site Conformations at RT than 100K

**DOI:** 10.1063/4.0000950

**Published:** 2025-10-27

**Authors:** Jonathan A Clinger

**Affiliations:** Baylor University, Waco, TX, USA

## Abstract

The polyamine biosynthetic pathway synthesizes the polyamines putrescine, spermidine, and spermine from ornithine and S-adenosyl methionine. These polyamines are important molecules in metabolism and are associated with growth and proliferation of cells and are upregulated in many cancers. Even though this pathway has been of interest to scientists and clinicians for medical intervention, FDA approved inhibitors are unavailable, as these enzymes have proven exceptionally challenging to drug. To gather more information about the structure and dynamics of these enzymes, we have collected multi-temperature crystallography data for ornithine decarboxylase, S-adenosyl methionine decarboxylase, and spermidine synthase. Our data show dynamic enzymes where the room temperature structures differ from standard cryo- crystallography structures, improving our understanding of these enzymes and yielding insights that may be used for rational drug design.

